# Genes Regulated in Metastatic Osteosarcoma: Evaluation by Microarray Analysis in Four Human and Two Mouse Cell Line Systems

**DOI:** 10.1155/2012/937506

**Published:** 2012-11-13

**Authors:** Roman Muff, Ram Mohan Ram Kumar, Sander M. Botter, Walter Born, Bruno Fuchs

**Affiliations:** Laboratory for Orthopedic Research, Balgrist University Hospital, Forchstrasse 340, 8008 Zurich, Switzerland

## Abstract

Osteosarcoma (OS) is a rare bone neoplasm that affects mainly adolescents. It is associated with poor prognosis in case of metastases formation. The search for metastasis predicting markers is therefore imperative to optimize treatment strategies for patients at risk and important for the search of new drugs for the treatment of this devastating disease. Here, we have analyzed by microarray the differential gene expression in four human and two mouse OS cell line systems consisting of parental cell lines with low metastatic potential and derivatives thereof with increased metastatic potential. Using two osteoblastic cell line systems, the most common OS phenotype, we have identified forty-eight common genes that are differentially expressed in metastatic cell lines compared to parental cells. The identified subset of metastasis relevant genes in osteoblastic OS overlapped only minimally with differentially expressed genes in the other four preosteoblast or nonosteoblastic cell line systems. The results imply an OS phenotype specific expression pattern of metastasis regulating proteins and form a basis for further investigation of gene expression profiles in patients' samples combined with survival analysis with the aim to optimize treatment strategies to develop new drugs and to consequently improve the survival of patients with the most common form of osteoblastic OS.

## 1. Introduction

Osteosarcoma (OS) is a rare but highly malignant neoplasm of bone that affects mainly young patients during the second decade of their lives. The survival of patients with localized disease has been improved by refinement of surgical techniques and by the introduction of neoadjuvant chemotherapy. However, the survival rate of patients that develop metastases remains to be low. The identification of proteins that are involved in OS progression and metastasis is therefore of immediate importance to develop new and improved treatment strategies.

The analysis of differentially expressed genes by microarray, comparing metastatic OS cell lines to parental cell lines with low metastatic potential, should help to identify common pathways or even a set of proteins that regulate OS tumor progression and metastasis. To our knowledge, four human and two mouse OS systems were developed that fulfill this requirement. Human metastatic LM5 and M132 cells were derived from parental SAOS and HUO9 cells, respectively, by *in vivo* selection in mice carried out by repeated tail vein injection of cells isolated from lung metastases [1, 2]. Human metastatic 143B cells were obtained by K-ras transformation of HOS [3] cells and human metastatic M8 cells by *in vitro* subcloning of parental MG63 cells as described [4]. Mouse metastatic LM8 and K7M2 cells were also selected *in vivo* from parental Dunn and K12 cells, respectively [5, 6]. Comparative microarray analyses were performed with HUO9/M132 [7], K12/K7M2 [8], and most recently with SAOS/LM7 and HOS/143B cells [9]. The results obtained in these studies imply that different sets of proteins are differentially expressed in each system and that different signaling pathways are involved in OS tumor progression. These studies identified ezrin as an important player in OS pathogenesis [8, 10].

OS is a heterogeneous disease. Diverse cell types originating from mesenchymal stem cells may be affected by genomic instability during different stages of differentiation [11, 12]. Histologically, most of the patients present with tumors with an osteoblastic (60–70%) phenotype, followed by chondroblastic and fibroblastic OS (both approximately 10%) [13]. Although there is no evidence for a cell type dependent propensity to form metastases in OS [13], different pathways involved in tumor progression in such diverse cell types appear likely. SAOS and Dunn cells are considered osteoblast-like cells or early osteoblasts as they express high alkaline phosphatase (ALPL) activity, possess parathyroid hormone (PTH) responsiveness, and produce mineralized extracellular matrix upon osteogenic induction *in vitro* ([5, 14], and this study). HUO9 are also described to be osteoblastic [2], but the relatively low ALPL activity observed in this study suggests that they are preosteoblastic. MG63 and K12 are considered fibroblastic [15, 16], and HOS have a mixed type of fibroblastic and epithelial-like morphology.

In this study we analyzed differentially expressed genes by microarray analyses in the four human OS cell line systems SAOS/LM5, HUO9/M132, HOS/143B, and MG63/M8 and the two mouse cell line systems Dunn/LM8 and K12/K7M2. Based on the enrichment of differentially regulated genes in common gene ontology (GO) terms, we identified 48 (17 up- and 31 downregulated) commonly regulated genes in OS metastasis in the two osteoblastic systems (SAOS/LM5 and Dunn/LM8), that were shared only at a limited number in the other four cell line systems. The possible role of some of the identified genes in osteoblastic tumor progression is discussed.

## 2. Materials and Methods

### 2.1. Cell Lines and Culture

SAOS (HTB-85), HOS (CRL-1543), and 143B (CRL-8303) cells were obtained from ATCC (Rockville, MD, USA). LM5 cells were kindly provided by E.S. Kleinerman (M.D. Anderson Cancer Center, Houston, TX, USA), HUO9 and HUO9-M132 (M132) cells by M. Tani (National Cancer Center Hospital, Tokyo, Japan), Dunn and LM8 cells by T. Ueda (Osaka University Graduate School of Medicine, Osaka, Japan), MG63 cells by G. Sarkar (Mayo Clinic, Rochester, MN, USA), and MG63-M8 (M8) cells by W. T. Zhu (Tongji Hospital, Huazhong University of Science and Technology, Wuhan, China). K12 and K7M2 cells were obtained from C. Khanna (National Cancer Institute, Bethesda, MD, USA), the former with permission from J. Schmidt (GSF, National Research Center for Environment and Health, Neuherberg, Germany) who established the K12 cells [16]. Cells were cultured in DMEM (4.5 g/L glucose)/F12 (1 : 1) medium supplemented with 10% heat-inactivated FCS in a humidified atmosphere of 5% CO_2_. Subconfluent cells were detached with trypsin/EDTA, centrifuged and cell pellets immediately frozen in liquid nitrogen, and stored at −80°C until RNA extraction.

### 2.2. RNA Extraction, Array Hybridization, and Analysis

Total RNA was isolated from frozen cell pellets of individual cell lines with TriReagent (Sigma-Aldrich, St. Louis, MO) as described [17]. The RNA was quantified by measuring the absorption at 260 and 280 nm in a UV-spectrophotometer. The integrity of the RNA was assessed by standard agarose gel-electrophoresis and using Bioanalyzer 2100. Complementary RNA preparation and array hybridization were performed by the Functional Genomics Center (Zurich, Switzerland) using Affymetrix Human Genome U133 Plus 2.0 (54675 probe sets) and Affymetrix Mouse Genome 430 2.0 (45101 probe sets) arrays. The gene expression signals ranged from 5–22000 and 6–31000 in the four human and two mouse systems, respectively. The distribution of gene expression levels (Log_2_) was similar in the two array types as exemplified for the SAOS/LM5 and Dunn/LM8 systems (Supplementary Figure 1 of the Supplementary Material available at doi:10.1155/2012/937506). Gene expression levels were arbitrarily set as low, intermediate, and high when values were <50, 50–300, and >300, respectively. By this criterion approximately 60% of the genes were expressed at a low level, 20% were expressed at intermediate, and the remaining 20% at high levels in both types of arrays. Quality control, RMA normalization of raw data, and statistical analysis were performed using RACE (http://race.unil.ch/). Gene ontology (GO) analysis was performed using GOEAST (http://omicslab.genetics.ac.cn/GOEAST/). We used Ingenuity Pathway Analysis (IPA) version 12710793, build 162830. In the first step of a so-called core analysis, the genes (molecules) that belong to the regulated probe sets are identified. Next, the cutoff fold-change was adjusted to select around 800 regulated molecules (to minimize noise, Ingenuity recommends that approximately 800 molecules, or less, are analyzed). These molecules are eligible for generating networks, where (in)direct and relationships between molecules are shown based on literature findings. The networks are limited to 35 molecules each in order to keep them to a usable size.

### 2.3. cDNA Synthesis and Real-Time PCR Analysis

cDNA was reverse transcribed from 1 *μ*g of total RNA with a high-capacity RNA-to-cDNA kit (Applied Biosystems, Foster City, CA) and the protocol provided by the manufacturer. Three independent RNA extracts from individual cell lines were reverse transcribed in a final volume of 20 *μ*L. Real-time PCR was carried out in a StepOnePlus Real-Time PCR System (Applied Biosystems) in 96-well plates. Intron-spanning primers were designed with NCBI Primer blast (http://www.ncbi.nlm.nih.gov/tools/primer-blast/) software (Supplementary Table 1) for amplification of cDNA sequences derived from selected genes. Five genes that were found upregulated and five genes that were found downregulated by microarray analysis in LM5 compared SAOS cells and GAPDH as a reference gene were analyzed. PCR from individual RT reactions was carried out in triplicates. cDNA equivalent to 30 ng of RNA and appropriate primers were added to Power SYBR Green PCR Master Mix (Applied Biosystems) and the samples preincubated at 50°C for 2 min and at 95°C for 10 min and then subjected to 40 cycles of incubation at 95°C for 15 s and at 60°C for 1 min. The threshold for Ct values was set to 0.325 and the obtained values were analyzed with the delta Ct (ΔCt) method. Mean Ct values calculated from triplicate PCR were normalized to mean Ct values determined for GAPDH gene transcripts as a measure for cDNA input. The presence of nonspecific amplification products in any of the PCR reactions was excluded by inspection of the melting curves of final PCR products. The data presented in Supplementary Figure 2 confirmed the upregulation of four and the downregulation of five genes as revealed by the microarray analysis.

### 2.4. Alkaline Phosphatase Activity, cAMP Stimulation, and Alizarin Red S Staining

Cell extraction and measurements of alkaline phosphatase (ALPL) activity, cAMP production stimulated by chicken parathyroid hormone-related peptide, the induction of extracellular matrix mineralization, and its visualization by Alizarin Red S staining were performed as described [14].

## 3. Results

### 3.1. Gene Expression Analysis Reveals Heterogeneity among Different Cell Line Systems and Differential Gene Expression in Low and High Metastatic Cell Lines

**Figure 1:**
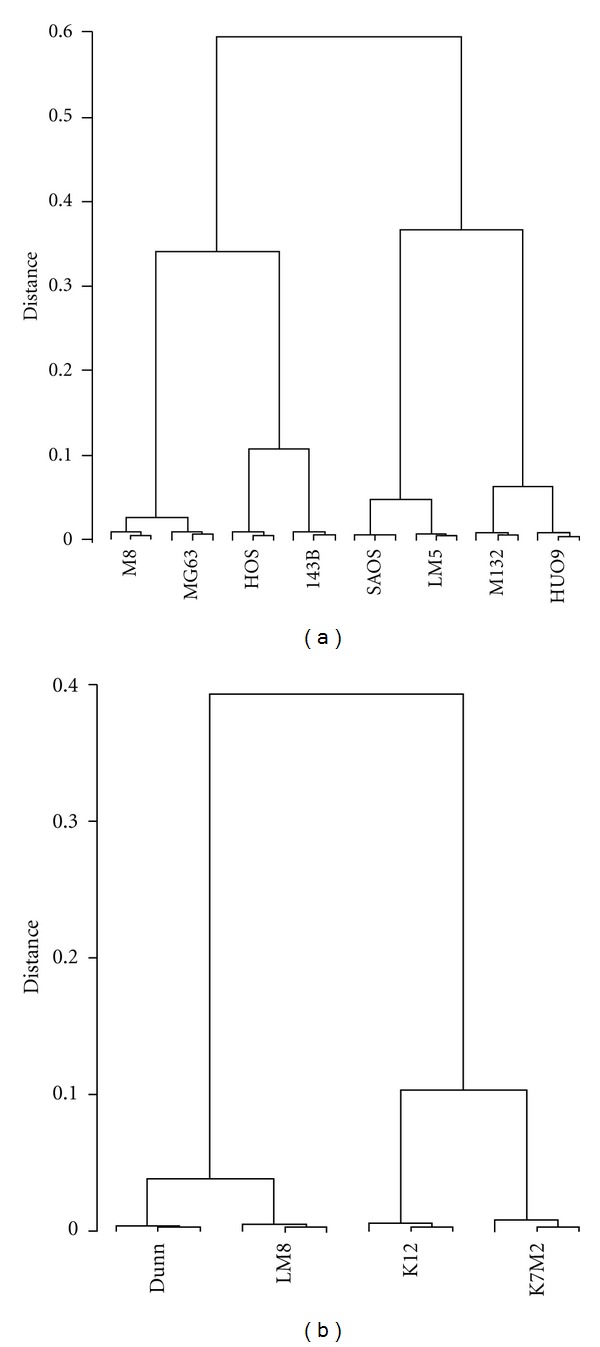
(a) Dendrogram of gene expression levels in the four human OS cell line systems. (b) Dendrogram of gene expression levels in the two mouse OS cell line systems. All probe sets on the arrays were included in the analysis.

Four human and two mouse osteosarcoma cell lines with low metastatic potential were compared to their high-metastatic derivatives for differential gene expression by microarray analysis. The four human systems with low and upregulated metastatic activity included the SAOS/LM5, HUO9/M132, HOS/143B, and MG63/M8 cells. The two mouse systems consisted of the Dunn/LM8 and K12/K7M2 cells. A comparison of gene expression levels in the four human systems revealed that each system clustered together, although the expression levels clearly differed in low versus high metastatic cell lines (Figure 1(a)). Interestingly, the two cell line systems that underwent *in vivo* selection of the cell line with increased metastatic potential (SAOS/LM5 and HUO9/M132) were clearly different from the cluster of the HOS/143B (Ki-ras transformation of HOS) system and the MG63/M8 system obtained by *in vitro* selection of M8 from MG63. Clustering of the two mouse systems with distinct gene expression levels in low and high metastatic cell lines was also observed (Figure 1(b)). The number of differently expressed genes (i.e., up- or downregulated >2-fold with a false discovery rate (fdr) of <0.01) in low versus high metastatic cell lines was highest in HOS/143B and K12/K7M2, lower in SAOS/LM5, HUO9/M132, and Dunn/LM8, and lowest in MG63/M8. Only 1% of total probe sets were differentially regulated in MG63/M8 whereas 2.5–3.6% (SAOS/LM5, HUO9/M132, and Dunn/LM8) and 6.7–8.3% were differentially regulated in the HOS/143B and K12/K7M2 systems, respectively. These findings are summarized in Table 1. Taken together, the results indicate remarkable heterogeneity among the different OS cell line systems and also variability in the number of genes potentially involved in malignancy progression in the different cell lines.

### 3.2. Plasma Membrane and Extracellular Matrix Proteins Involved in Binding, Cell Migration, Angiogenesis, and Apoptosis Are Differentially Expressed in Tumor Progression

A gene ontology (GO) analysis of metastasis-regulated (>2-fold; fdr < 0.01) genes in all six cell systems revealed an enrichment (fdr < 0.00001) at the most general level 1 (Table 2). Here, 5 out of 18 “cellular component,” 5 out of 20 “molecular function”, and 18 out of 32 “biological process” GO terms were significantly enriched, again with a large variability among the different cell systems. For the term “cellular component,” genes belonging to the terms “cell” and “extracellular region” were enriched in all cell systems, followed by “extracellular matrix” (4/6) and “membrane” (3/6). For the term “molecular function” only genes belonging to the term “binding” were enriched in all cell systems. The greatest variability was observed in the term “biological process.” Here, enrichment was observed in all cell systems in terms of “biological regulation,” “multicellular organismal process”, “developmental process,” and “biological adhesion,” followed by “cellular process” (5/6), “response to stimulus” (4/6), and “signaling” (4/6). In eleven additional terms enrichment was observed to different degrees in the six cell line systems. Despite the observed heterogeneity, the results imply that aberrantly expressed binding proteins in the plasma membrane and the extracellular matrix that control the regulation of developmental processes and cellular adhesion are involved in tumor progression in mice and humans.

A GO analysis at higher and more specific levels, and with significant nodes between levels and down to the level 1, revealed an aberrant regulation of diverse biological processes in the different cell systems (Supplementary Table 2). In the SAOS/LM5 cell line system, genes involved in cardiovascular development and neurogenesis were aberrantly expressed. In the HUO9/M132 system, heart development was also affected together with ureteric bud morphogenesis and genes involved in angiogenesis and cell migration were highly regulated. In the HOS/143B system, angiogenesis and cell migration were also deregulated. In addition, apoptosis and integrin-mediated cell-cell adhesion were affected. Genes involved in ERK1 and -2 kinase, MAP kinase, SMAD, and TGF*β* signaling and in ureteric bud, prostate gland, blood vessel and lung alveolus development, neurogenesis, and blood coagulation were also significantly enriched in respective GO terms. In the MG63/M8 system only genes involved in axon regeneration, cell surface receptor signaling, and intracellular protein kinase activity were differentially regulated. In the Dunn/LM8 system, genes involved in the regulation of cell migration and apoptosis and extracellular matrix organization together with epithelium, neuron, blood vessel, and chondrocyte differentiation were enriched in respective GO terms. Migration and angiogenesis were affected in the K12/K7M2 system together with altered ovarian follicle, mammary gland duct and salivary gland morphogenesis, and axonogenesis. Taken together, although no common biological processes were revealed in the six cell line systems, deregulation of cell migration (4/6), angiogenesis (3/6) and apoptosis (2/6) may contribute to tumor progression. The affected developmental processes were mainly neurogenesis (5/6), cardiovascular (4/6), and the reproductive system development.

In addition to GO, we also analyzed these data through the use of Ingenuity Pathways Analysis (IPA; Ingenuity Systems, http://www.ingenuity.com). The analysis confirmed all cell systems to be associated with cancerous processes (ranked as nr. 1 bio function “diseases and disorders” for all cell systems) (Supplementary Table 3). Also pathways involved in cellular movement (ranked nr. 1 in all cell systems, except for LM5) and cardiovascular development and function (ranked nr. 1 in 5 out of 6 cell systems, ranked nr. 3 in LM5), with vasculogenesis/angiogenesis pathways prominently activated, were found to be enriched. These results therefore confirm the results obtained in the GO analysis.

### 3.3. The SAOS/LM5 and Dunn/LM8 Cell Line Systems Are Representative for Osteoblastic OS

In humans, approximately two thirds of OS patients present with an osteoblastic tumor phenotype. We therefore searched for osteoblastic marker gene expression in our microarray data (Table 3) (for review see [18]). The transcription factor SOX9, a marker for osteoblast progenitor cells but not of mature osteoblasts, was expressed at low to intermediate levels in all cell lines, except in 143B, where it was upregulated 20-fold in comparison to HOS cells. RUNX2 (Runt-related transcription factor 2, also known as CBAF1), an early osteoblast differentiation transcription factor which is also required at lower levels for proper mature osteoblast function, was expressed at high levels in the Dunn/LM8, SAOS/LM5, HUO9/M132, and K12/K7M2 systems and at intermediate levels in the HOS/143B system. In the MG63/M8 system it was upregulated 4-fold from low to intermediate levels in M8 compared to MG63. OSX (osterix), a transcription factor that acts downstream of RUNX2, was expressed at intermediate to high levels in Dunn/LM8, SAOS/LM5, and HUO9/M132 cells. Low expression was observed in K12/K7M2, HOS/143B and MG63/M8 cells. COL1A1 (collagen 1), SPP1 (osteopontin, bone sialoprotein 1), and IBSP (bone sialoprotein 2) are produced by maturing and mature osteoblasts. COL1A1 was expressed at high levels in all cell lines except in 143B, where it was downregulated 145-fold compared to HOS. SPP1 was expressed at high levels in the Dunn/LM8, HUO9/M132, and K12/K7M2 systems and at low levels in the SAOS/LM5 and MG63/M8 systems. In HOS/143B it was upregulated 17-fold from low to intermediate levels in 143B compared to HOS cells. IBSP was only expressed at high levels in the Dunn/LM8 and SAOS/LM5 systems and was low in all other cell line systems. ALPL (liver/bone/kidney alkaline phosphatase) and PTH1R (parathyroid hormone receptor 1) are expressed by late osteoblast-like cells and mature osteoblasts. ALPL was expressed at high levels only in the Dunn/LM8 and SAOS/LM5 systems and was low in all other cell lines, except in the HOS/143B system where it was upregulated 10-fold from low to intermediate levels in HOS compared to 143B cells. PTH1R was expressed at intermediate to high levels in the Dunn/LM8, SAOS/LM5, and HUO9/M132 cell lines and at low levels in the K12/K7M2 and MG63/M8 cell lines. Similar to ALPL, PTH1R was upregulated 3-fold from low to intermediate levels in HOS compared to 143B. In summary, only the Dunn/LM8 and SAOS/LM5 cell lines exhibited gene expression characteristics of osteoblast-like cells. HUO9/M132 and K12/K7M2 cell lines appear to be osteoblastic precursors that were stalled in the osteoblastic differentiation process at an intermediate step, whereas the HOS/143B and MG63/M8 lines were stalled at an early stage.

**Figure 2:**
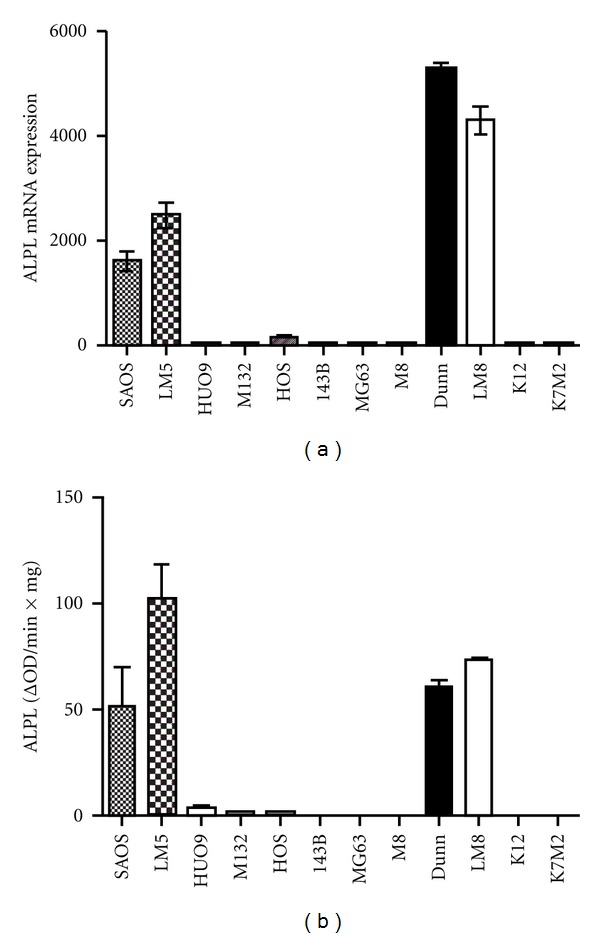
(a) RMA normalized ALPL (human: 215783_s_at; mouse: 1423611_at) mRNA expression. (b) ALPL enzyme activity.

The high ALPL gene expression in Dunn/LM8 and SAOS/LM5 was confirmed by measuring the enzyme activity in cell extracts (Figure 2). Although minimal enzyme activity was detectable in HUO9, M132, and HOS cells, it was 25-fold lower compared to Dunn/LM8 and SAOS/LM5, whereas no enzyme activity was measurable in the other cell lines. The osteoblast-like phenotype of SAOS/LM5 cells, including PTH (parathyroid hormone) stimulated cAMP production and the formation of mineralized extracellular matrix has already been described [14]. In the present study, PTH stimulated cAMP production also in Dunn (129 ± 27-fold; *n* = 4, *P* < 0.02) and LM8 cells (111 ± 17-fold; *n* = 3, *P* < 0.03) and mineralization of extracellular matrix upon induction with ascorbic acid, beta-glycerophosphate, and dexamethasone was also observed (not shown). In summary, the enzyme activity profile of the Dunn/LM8 and SAOS/LM5 cell lines paralleled the expression profile obtained with gene expression analysis, confirming their osteoblast-like properties at a functional level.

### 3.4. The Osteoblastic OS Cell Line Systems Present with an Enriched Set of Commonly Regulated Genes as Potential Targets for OS Treatment

In face of a similar number of regulated metastasis-related genes in Dunn/LM8 and SAOS/LM5 cell line systems (Table 1) and a similar enrichment pattern in GO terms (Table 2), we investigated if both systems had commonly regulated genes that might contribute to tumor progression and metastasis specific for osteoblastic OS. First, regulated (>2-fold; fdr < 0.01) probe set lists in both systems were separated in up- and downregulated probe set lists in metastatic versus parental cell lines. Second, separated up- and downregulated probe set lists were analyzed in GO for commonly enriched GO terms at the levels indicated in Supplementary Table 4. Upregulated probe sets were enriched in both systems in four GO terms and downregulated probe sets in seven GO terms. The GO probe set lists were then analyzed for commonly regulated genes (average of approximately 1.4 probe sets/gene) in both cell line systems and the results are summarized in Table 4. Using this strategy, we found 48 genes to be unidirectionally regulated in both Dunn/LM8 and SAOS/LM5 cell line systems, of which 17 genes were upregulated and 31 genes were downregulated. Real-time PCR analysis confirmed the upregulation of 4 out of 5 and the downregulation of 5 out of 5 genes that were selected based on the results of the microarray analysis of the SAOS/LM5 cell line system (Supplementary Figure 2). This is well within the range we previously observed by PCR and/or Western blot analysis for other gene products in this and other cell line systems (not shown).

From the relative frequencies given in Table 1, we can calculate the number of genes to be up- or downregulated in both systems by chance to be between 4 to 6, when analyzing the total datasets. As we have restricted our analysis to genes only enriched in some common GO terms in both cell line systems, the number of commonly regulated genes (48) by far exceeds the number of genes that would be selected by chance. Therefore, these genes can be considered to be relevant in osteoblastic OS metastasis. In the remaining four cell line systems, we found that of the 17 upregulated genes in our osteoblastic gene panel 1, 4, 4, and 0 genes were also upregulated in M132, K7M2, 143B, and M8, respectively, but similar numbers of genes were found to be regulated in the opposite direction as well. Likewise, of the 31 genes found downregulated 3, 5, 9, and 0 genes were also downregulated in M132, K7M2, 143B, and M8, respectively, but again similar numbers of genes were regulated in the opposite direction. Given the fact that in 143B, and K7M2 the total number of regulated genes was double the number found in M132, LM5, and LM8 cells, whereas compared to M8 cells the total number was only half (Table 1), it is likely that these genes may be commonly regulated by chance and do not necessarily contribute to tumor progression in these preosteoblast or nonosteoblastic systems.

We next looked in IPA if the 48 genes in our selected osteoblastic panel belonged to a specific pathway. This analysis revealed that half of the selected genes (24 out of 48) are related to inhibition of cell death, making it the top molecular and cellular function. Pathways related to cellular growth and proliferation were also stimulated, with the functions “proliferation of cells” and “proliferation of tumor cell lines” significantly enriched. The function “colony formation of (tumor) cells” was inhibited. Surprisingly, we also found the function “migration of cells” to be inhibited, most notably by inhibition of SERPINE2, although the expression of PAX3 (included in the same function and associated with increased migration) was found to be increased, indicating some variability in this function.

Three networks were found to be significantly regulated, of which “cell morphology, cellular assembly and organization, cellular function and maintenance” obtained the highest score with 21 regulated molecules of a total of 35 (Supplementary Figure 3), followed by “cell death, cell morphology, cell-to-cell signaling and interaction” (13 regulated out of 35) and “free radical scavenging, small molecule biochemistry, nervous system development and function” (12 regulated out of 35). These last two networks were found to be overlapping.

One interesting feature of IPA is to identify upstream regulators, such as transcription factors, that may not show an expressional change (and are therefore not included in the analyzed gene list), but are nevertheless likely to be activated based on a change in target gene expression. Using this approach, RUNX2 was identified as one of the top activated transcription factors, because its direct targets COL24A1, TPM1, HAPLN1, and ACTA2 were present among the common regulated genes. Likewise, we identified MEIS2 (targets ETV1, PAX3, PAX6) to be significantly active. This transcription factor has not been associated to OS metastasis before, but was earlier found to be regulated in lung cancer [29], ovarian cancer [30], thyroid cancer [31], and prostate cancer [32].

## 4. Discussion

Metastasis-related microarray analyses have been performed using parental low metastatic HUO9, K12; HOS and SAOS cells and metastatic derivatives thereof [7–9]. Seven metastasis-related genes were described for the HUO9/M132 system and we found all these genes also differentially regulated in the same direction in our array. From the eleven genes described to be metastasis related in the K12/K7M2 system we found an overlap of 70%. This was also seen for the ten top upregulated genes in the HOS/143B cell line system. A 50% overlap was also observed when we compared our SAOS/LM5 system with the SAOS/LM7 system [9]. Generally, a high overlap in differentially regulated genes was observed when different microarray analyses were compared. 

Several biological processes involved in tumor progression, such as proliferation, motility, invasion, immune surveillance, adhesion, and angiogenesis, have been identified in the K12/K7M2 system [8]. We have also found enrichment in the GO terms “proliferation,” “motility,” “adhesion,” and “angiogenesis” although at lower levels than those listed in Supplementary Table 2. This again confirms a high overlap between the two independent analyses of this system. The former study identified ezrin as the most prominent protein involved in OS progression. We therefore looked for ezrin expression in our six cell line systems. Indeed, ezrin was overexpressed 70-fold in K7M2 compared to K12 cells. However, in LM8 and 143B cells, ezrin was downregulated 1.8- and 2.7-fold compared to the respective parental cells and in the MG63/M8 system it was not regulated. In LM5 and M132 cells it was found only minimally upregulated (<2 fold) compared to levels in corresponding parental cells. Thus, based on these *in vitro* gene expression analyses, ezrin may only play a role in OS progression in a subgroup of OS tumors.

The comparison of gene expression levels in six cell line systems revealed a high heterogeneity, illustrated by the more than 7-fold difference in the total number of differentially expressed probe sets. The enrichment in common GO terms was also low. This indicates that the cellular mechanisms involved in tumor progression differ in each system. This is in line with the hypothesis that genomic instability (chromothripsis) arising at different steps of osteoblastic commitment is a cause of OS oncogenesis [12]. In the SAOS/LM5 and Dunn/LM8 cell line systems, in which we confirmed the osteoblast-like nature, the still high RUNX2 expression makes it likely that chromothripsis took place at an early-osteoblast stage [11]. In addition, these two osteoblast-like cell line systems had the highest overlap in the number of regulated metastasis relevant genes and in GO enrichment. An analysis of a subset of differentially expressed genes that were enriched in common GO terms revealed common regulation of 48 genes in the osteoblastic cell line systems, exceeding the gene number expected from probability calculations. Thus, these genes are likely to contribute to tumor progression or chromothripsis survival in osteoblastic OS. According to the chromothripsis hypothesis, different sets of proteins are then likely to contribute to survival in different cancer cell types or in osteoprogenitor cells at different developmental stages as observed in this study.

Seventeen genes were commonly upregulated in the two metastatic osteoblastic cell lines LM5 and LM8 compared to the corresponding parental cell lines. The relevance of some of these genes is now discussed with reference to published literature on cancer in general and on OS in particular.

SERPINE2 (protease nexin 1) is a secreted serine protease inhibitor that inhibits among others the blood coagulation factors Xa and XIa, thrombin, tPA and uPA [33]. Here, SERPINE2 was upregulated in metastasis in four out of six cell line systems and remarkably downregulated in one system. Increased expression of a protease inhibitor would rather be expected to reduce invasiveness and hence malignant progression, which is also indicated by our IPA analysis. Indeed, overexpression of SERPINE2 in prostate cancer cells reduces their invasion capacity [34], and in experimental OS, inhibition of uPAR pathway resulted in decreased lung metastasis [19]. In pancreatic tumors, on the other hand, SERPINE2 promotes extracellular matrix production and local invasion *in vivo* [35]. SERPINE2 expression is elevated in colorectal cancer, correlates with tumor grade and its silencing reduces anchorage-independent growth, migration, and tumor formation [36, 37]. SERPINE2 expression is also increased in breast cancer [38]. SERPINE2 promotes lymph node metastasis of testicular cancer [39] and SERPINE2 expression also correlates with selective lung metastasis in breast cancer [40] and lymph node metastasis of oral squamous cell carcinoma [41]. Metastasis of lung cancer cells towards bone is also associated with increased SERPINE2 expression [42]. Thus, SERPINE2 has a dual role in cancer progression and its role in OS progression, probably uPA/uPAR independent, may be further investigated.

FHOD3 (formin homolog overexpressed in spleen 2) is a member of a family of proteins involved in actin assembly and located in the cytoplasm. FHOD3 is predominantly expressed in heart and regulates sarcomere organization in striated muscles [43]. Although there is no evidence yet for a role of FHOD3 in other cancer types, the downregulation of FHOD1 (Swiss-Prot Q9Y613), another member of the family, reduces migration and invasion of breast cancer cells *in vitro* [44]. Interestingly, in the present study, FHOD3 was found upregulated in four metastatic cell lines.

PAX3 (paired box protein Pax-3) and PAX6 (paired box protein Pax-6) are nuclear transcription factors involved in the development of many tissues and the role of PAX3 in rhabdomyosarcoma and malignant melanoma is discussed [45, 46]. PAX3 is also expressed in most Ewing's sarcoma samples [47]. PAX3 was exclusively found overexpressed in the present study in metastatic osteoblastic OS, whereas PAX6 was overexpressed in four cell line systems. PAX3 overexpression in SAOS *in vitro* induces mesenchymal-epithelial transition (MET) and increases cell motility (IPA analysis of LM5–LM8 common regulated genes and [21, 22]), and therefore PAX3 expression analysis during osteoblastic tumor progression *in vivo* deserves further examination.

DLX4 (homeobox protein DLX-4) is a nuclear transcription factor that is overexpressed in several cancer types [48–51]. DLX4 suppresses the antiproliferative effect of TGF-*β* [52] and has an antiapoptotic function [53]. Upregulation of DLX4 increases the metastatic potential of breast cancer cells *in vitro* and *in vivo* [49, 54] and of prostate adenocarcinoma [51], and its expression correlates with advanced disease stage in ovarian cancer [50]. Here, DLX4 was also found upregulated in three cell line systems, but it was downregulated in 143B cells. To this end, downregulation of DLX4 correlates with increased metastatic potential *in vitro* and *in vivo* in lung cancer [55].

FOXQ1 (forkhead box protein Q19) is a nuclear transcription factor involved in mammary epithelial cell differentiation and in epithelial-mesenchymal transition (EMT) in breast cancer cells [56–58]. Its expression correlates with cancer cell aggressiveness *in vitro *and with lung metastasis *in vivo* in mice. FOXQ1 is also overexpressed in colorectal cancer where it increases tumorigenicity by its angiogenic and antiapoptotic effects [59]. Here, FOXQ1 was also found overexpressed in metastatic osteoblastic OS cells. Interestingly, factors that control EMT, such as FOXQ1, and mesenchymal-epithelial transition (MET), such as PAX3 were overexpressed in OS metastasis *in vitro*.

LOX (lysyl oxidase; EC 1.4.3.13) is a secreted enzyme that is involved in extracellular matrix (collagen and elastin) crosslinking and is also produced by mature osteoblasts [60]. Here, LOX expression was high in all cell lines investigated *in vitro* except in SAOS/LM5 cells (not shown). Previously we showed increased LOX expression in MG63 cells compared to fetal osteoblasts indicating that LOX might be related to OS formation [20]. Despite the fact that LOX expression did not correlate here with the osteoblastic phenotype, LOX expression was upregulated during metastatic progression in the two osteoblastic cell line systems, but downregulated in three out of four nonosteoblastic cell line systems. Tumor suppressor as well as metastasis promoting functions of LOX have been described in several cancer types [61]. Analyzing LOX protein expression in OS patients should answer the question whether LOX expression has a dual role in OS progression depending on the cellular background.

PCBD1 (pterin carbinolamine dehydratase or dimerization cofactor of hepatocyte nuclear factor (HNF) 1-alpha; EC = 4.2.1.96) is involved in tetrahydrobiopterin recycling, a cofactor used in the degradation of the amino acid phenylalanine. It regulates the transcriptional activity (i.e., homodimerization) of HNF and is located in the cytoplasm and inside the nucleus. It is overexpressed in colon cancer and in melanoma [62, 63] but to our knowledge has so far not been described in tumor progression. Here PCBD1 was exclusively overexpressed in osteoblastic metastatic OS cells.

EHF (epithelium-specific Ets transcription factor 3) is a nuclear transcription factor involved in breast cancer tumorigenesis [64] and is a marker for poor survival in ovarian carcinoma [65]. Here, EHF was exclusively overexpressed in the two osteoblastic metastatic cell lines.

Thirty-one genes were commonly downregulated in metastatic osteoblastic OS cell lines. The relevance of some of the genes in OS progression is now discussed.

CCDC80 (downregulated by oncogenes protein 1) is a secreted tumor suppressor protein that facilitates the apoptotic cascade [66] and mediates growth inhibition in colon and pancreatic cancer [67]. CCDC80 was the gene that was commonly regulated in all cell systems investigated except MG63/M8. Downregulation of this tumor suppressor gene deserves further investigation in OS.

DAB2 (disabled homolog 2), a protein of clathrin coated pits, is a negative regulator of the Wnt/*β*-catenin pathway and therefore a putative tumor suppressor [68]. DAB2 is underexpressed in tumors compared to normal tissue and correlates with the malignant phenotype of lung cancer, urothelial carcinoma, squamous cell carcinoma, nasopharyngeal carcinoma, and esophageal squamous carcinoma [69–73]. DAB2 expression is also reduced in breast cancer, which results in up-regulation of TGF*β*2 that promotes EMT transition [74, 75]. In ovarian cancer low DAB2 levels correlate with poor outcome, but very low levels that inhibit EMT correlate with a better prognosis [76]. Here, DAB2 was downregulated in four metastatic OS cell lines.

TGFB2 (transforming growth factor beta-2) is a secreted protein that can act as a tumor suppressor in early stages of tumorigenesis or as a metastasis promoting factor in advanced cancers (for review see [77]). In OS, elevated TGFB3 expression correlates with poor survival [23]. In the same study with only 25 patients TGFB2 was not predictive, but low TGFB2 showed a trend towards poor survival. The here observed downregulation of TGFB2 in 4 metastatic cell lines should therefore be investigated further.

SLC1A3 (excitatory amino acid transporter 1) was found downregulated in metastasis in four cell line systems contained in this study. This is in contrast with findings in tumors of OS patients, where high expression of SLC1A3 was associated with a poor prognosis [24].

OSMR (oncostatin-M-specific receptor subunit beta or interleukin-31 receptor subunit beta) is a receptor for oncostatin M (OSM; Swiss-Prot P13725), a member of the interleukin-6 cytokine family. OSM inhibits cell proliferation via the JAK/STAT pathway in a number of tumor cells (for review see [78]), including OS [15] and chondrosarcoma [79]. In OS, OSM sensitizes cells to apoptosis [25], and its overexpression was found to reduce primary tumor growth and lung metastasis formation *in vivo* in mice [26]. These data, and the loss of OSMR in metastatic cell lines as observed in this study in four of the six analyzed cell line systems, point to an important role of OSM and OSMR in inhibition of OS tumor progression. On the other hand, OSM enhanced the *in vitro* metastatic activity in a subset of canine and human OS cells [80]. To this end, it is interesting to note that OSM had different effects on osteoblastic differentiation, depending on the maturation stage [15].

TPM1 (tropomyosin alpha-1 chain) is an actin filament-binding protein with diverse biological actions including malignant transformation (for review see [81]), and it is considered as a tumor suppressor gene. TPM1 was found downregulated in colorectal cancer compared to normal tissue [82]. In breast cancer cells, loss of TPM1 conferred anoikis resistance [83] and overexpression of TPM1 suppressed anchorage-independent growth [84]. In this study, TPM1 was found downregulated in three metastatic OS cell lines.

DEPDC6 (DEP domain-containing mTOR-interacting protein or DEPTOR) is a negative regulator of mTOR signaling and is therefore expected to be a tumor suppressor [85]. However, high DEPDC6 levels have been correlated with poor prognosis in myeloma and hepatocellular carcinoma [86, 87], indicating an oncogenic role. The here observed downregulation in three metastatic OS cell lines deserves therefore further examination.

PHLDA1 (pleckstrin homology-like domain family A member 1 or apoptosis-associated nuclear protein or TSSC3) is an apoptosis regulator. Downregulation of this protein is associated with metastasis progression in breast cancer and in melanoma [88, 89]. In OS, overexpression of TSSC3 induced apoptosis *in vitro* and reduced tumor growth *in vivo* in mice [27, 28]. The results are consistent with downregulation of PHLDA1 in metastatic LM5 and LM8 cells.

Interestingly, some of the discussed proteins are involved in EMT and MET (PAX3, FOXQ1, and DAB2), TGF/Wnt/*β*-catenin signaling (DLX4, DAB2, and TGFB2), JAK/STAT (OSMR) or mTOR (DEPDC6) pathways. In another microarray study, the TGF/Wnt/*β*-catenin pathway has also been associated with increased OS metastatic activity [9].

In conclusion, we have identified a significant number of differentially expressed genes in low and highly metastatic osteoblastic OS cell lines. These genes should be considered for further evaluation as key players in tumor progression in osteoblastic OS, the predominant phenotype of the disease.

## Supplementary Material

Supplementary Figure S1: (a) Distribution of mean log_2_ gene expression levels in SAOS/LM5 system. (b) Distribution of mean log_2_ gene expression levels in Dunn/LM8 system.
Supplementary Figure S2: Quantification of gene expression by real-time PCR in SAOS (open bars) and LM5 (black bars) cells. (a) Up-regulated and (b) down-regulated genes in metastatic LM5 cells compared to non-metastatic parental SAOS cells. GAPDH was used as a reference gene.
Supplementary Figure S3: Top network identified by Ingenuity Pathway analysis after analysis of 48 commonly regulated genes in SAOS/LM5 and Dunn/LM8 cell systems. Red indicates down-regulation (fold change >2, fdr < 0.01) in both cell systems, green indicates up-regulation, with the color intensity indicating the degree of up- or down-regulation.
Supplementary Table S1: PCR primers used for validation of microarray data, shown in Supplementary Figure S2.
Supplementary Table S2: Number of regulated (>2-fold; fdr < 0.01) probe sets enriched (fdr < 0.00001) in GO analysis at levels higher than 1, but with significant nodes down to level 1.
Supplementary Table S3: Ranking of top bio functions following IPA analysis. For each cell system, a cut-off was chosen such that comparable amounts of molecules were analyzed (in MG63/M8, a lower number of probe sets -521- were found to be significantly regulated, corresponding to a lower number -364- of recognized molecules). The top-5 bio functions, divided into the subcategories “diseases and disorders”, “molecular and cellular function” and “physiological system development and function” were compared among cell systems with the order displayed. Functions that were most commonly observed are shown first for each subcategory.
Supplementary Table S4: GO terms commonly up- or down-regulated in metastasis in the SAOS/LM5 and Dunn/LM8 cell line systems.

## Figures and Tables

**Table 1:** Number of regulated (>2-fold; fdr < 0.01) probe sets in human and mouse OS cell line systems.

System	Total (%)^a^	Up (%)^b^	Down (%)^b^
Human			
SAOS/LM5	1351 (2.5)	668 (49)	683 (51)
HUO9/M132	1975 (3.6)	1001 (51)	974 (49)
HOS/143B	3652 (6.7)	1690 (46)	1962 (54)
MG63/M8	521 (1.0)	292 (56)	229 (44)
Mouse			
Dunn/LM8	1217 (2.7)	494 (41)	723 (59)
K12/K7M2	3749 (8.3)	1769 (47)	1980 (53)

^a^Percent of the total number of probe sets represented by the human (54675) and mouse arrays (45101); ^b^percent of the total number of regulated probe sets.

**Table 2:** Number of regulated (>2-fold; fdr < 0.01) probe sets enriched (fdr < 0.00001) in GO terms level 1.

	SAOS/LM5	HU09/M132	HOS/143B	MG63/M8	Dunn/LM8	K12/K7M2
Cellular component						
Cell	884	1244	2519	362	780	2423
Membrane	486			206	475	
Extracellular region	196	266	403	96	218	365
Extracellular matrix	78		143		88	134
Synapse			112			
Molecular function						
Binding	850	1219	2435	343	805	2391
Molecular transducer activity		189	302			
Nucleic acid binding transcription factor activity		138	249			
Receptor activity		206				
Catalytic activity						1062
Biological process						
Cellular process	716	1017	2028		638	1858
Biological regulation	517	793	1537	250	522	1378
Response to stimulus		589	1074		376	1010
Multicellular organismal process	400	562	1055	186	369	846
Developmental process	345	490	931	159	351	768
Signaling		399		139	227	567
Biological adhesion	110	145	260	53	70	193
Growth	43		78			85
Locomotion		107				148
Cellular component organization or biogenesis			642			558
Localization			643			
Cell proliferation			133			
Rhythmic process			57			
Reproduction					83	
Immune system process					92	
Multiorganism process						129
Metabolic process						1132
Death						168

**Table 3:** Microarray mRNA expression levels of osteoblastic marker genes.

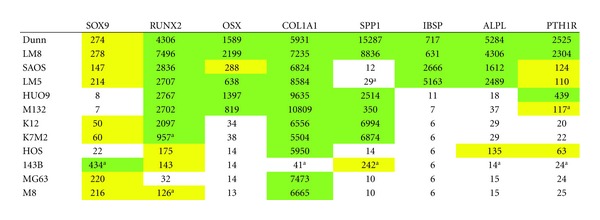

Results are means of the probe sets listed below and analyzed in triplicates. ^a^ >2-fold (*P* < 0.05) versus parental cell line. White, low expression (<50): yellow, intermediate expression (50–300); green, high expression (>300). Probe sets (human/mouse): SOX9 (202935_s_at/1451538_at) RUNX2 (232231_at/1424704_at); OSX (1552340_at/1418425_at); COL1A1 (1556499_s_at/1423669_at); SPP1 (209875_s_at/1449254_at); IBSP (236028_at/1417484_at); ALPL (215783_s_at/1423611_at); PTH1R (205911_at/1417092_at).

**Table 4:** Commonly up- and downregulated genes in metastasis.

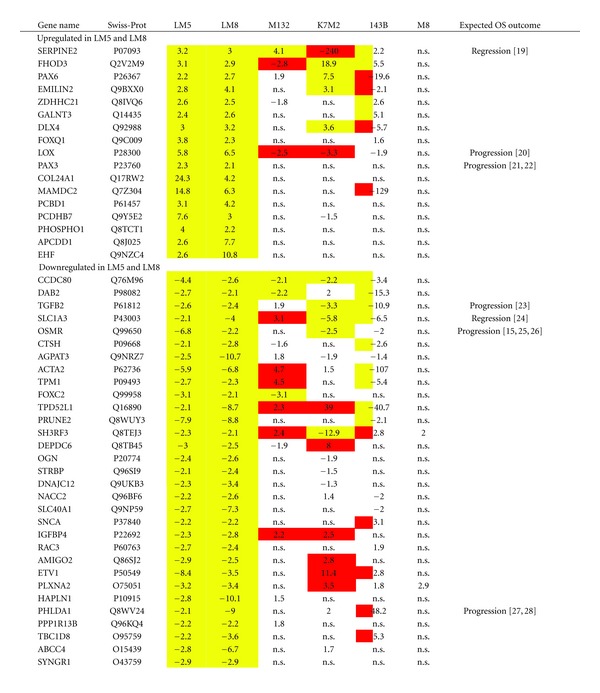

Positive numbers are upregulated genes and negative numbers downregulated genes in metastatic versus parental cell lines. For LM5 and LM8: >±2-fold (fdr < 0.01). For M132, K7M2, 143B, and M8: yellow (>±2-fold (fdr < 0.01) in the same direction as LM5 and LM8; white (fdr < 0.01); red (>±2-fold (fdr < 0.01) in the opposite direction as LM5 and LM8; n.s., not significant (fdr > 0.01). Gene names and Swiss-Prot (http://www.expasy.org/) numbers refer to human proteins.
