# Supplementary material for: Genes Regulated in Metastatic Osteosarcoma: Evaluation by Microarray Analysis in Four Human and Two Mouse Cell Line Systems
Source: Sarcoma. 2012 Nov 13;2012:937506. doi: 10.1155/2012/937506 (PMC3504467; doi:10.1155/2012/937506)
Supplement: Supplementary file 1 — Supplementary Figure S1: (a) Distribution of mean log2 gene expression levels in SAOS/LM5 system. (b) Distribution of mean log2 gene expression levels in Dunn/LM8 system. Supplementary Figure S2: Quantification of gene expression by real-time PCR in SAOS (open bars) and LM5 (black bars) cells. (a) Up-regulated and (b) down-regulated genes in metastatic LM5 cells compared to non-metastatic parental SAOS cells. GAPDH was used as a reference gene. Supplementary Figure S3: Top network identified by Ingenuity Pathway analysis after analysis of 48 commonly regulated genes in SAOS/LM5 and Dunn/LM8 cell systems. Red indicates down-regulation (fold change >2, fdr < 0.01) in both cell systems, green indicates up-regulation, with the color intensity indicating the degree of up- or down-regulation. Supplementary Table S1: PCR primers used for validation of microarray data, shown in Supplementary Figure S2. Supplementary Table S2: Number of regulated (>2-fold; fdr < 0.01) probe sets enriched (fdr < 0.00001) in GO analysis at levels higher than 1, but with significant nodes down to level 1. Supplementary Table S3: Ranking of top bio functions following IPA analysis. For each cell system, a cut-off was chosen such that comparable amounts of molecules were analyzed (in MG63/M8, a lower number of probe sets -521- were found to be significantly regulated, corresponding to a lower number -364- of recognized molecules). The top-5 bio functions, divided into the subcategories “diseases and disorders”, “molecular and cellular function” and “physiological system development and function” were compared among cell systems with the order displayed. Functions that were most commonly observed are shown first for each subcategory. Supplementary Table S4: GO terms commonly up- or down-regulated in metastasis in the SAOS/LM5 and Dunn/LM8 cell line systems. [file 937506.f1.docx]

SUPPLEMENTARY FIGURE 1


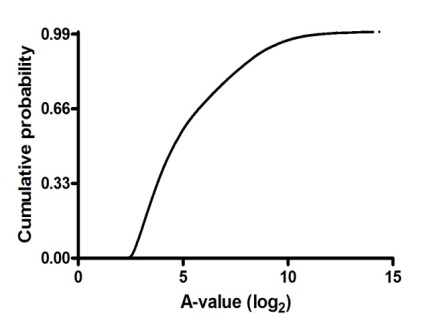

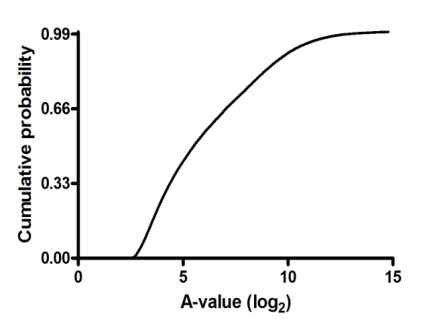


(a) (b)

SUPPLEMENTARY FIGURE 1: (a) Distribution of mean log_2_ gene expression levels in SAOS/LM5 system. (b) Distribution of mean log_2_ gene expression levels in Dunn/LM8 system.

SUPPLEMENTARY FIGURE 2


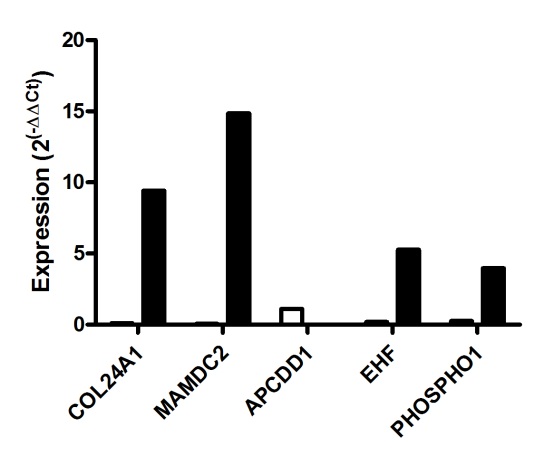

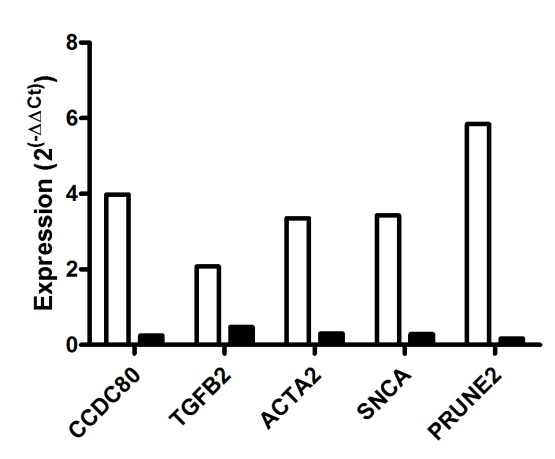


(a) (b)

SUPPLEMENTARY FIGURE 2: Quantification of gene expression by real-time PCR in SAOS (open bars) and LM5 (black bars) cells. (a) Up-regulated and (b) down-regulated genes in metastatic LM5 cells compared to non-metastatic parental SAOS cells. GAPDH was used as a reference gene.

SUPPLEMEMTARY FIGURE 3


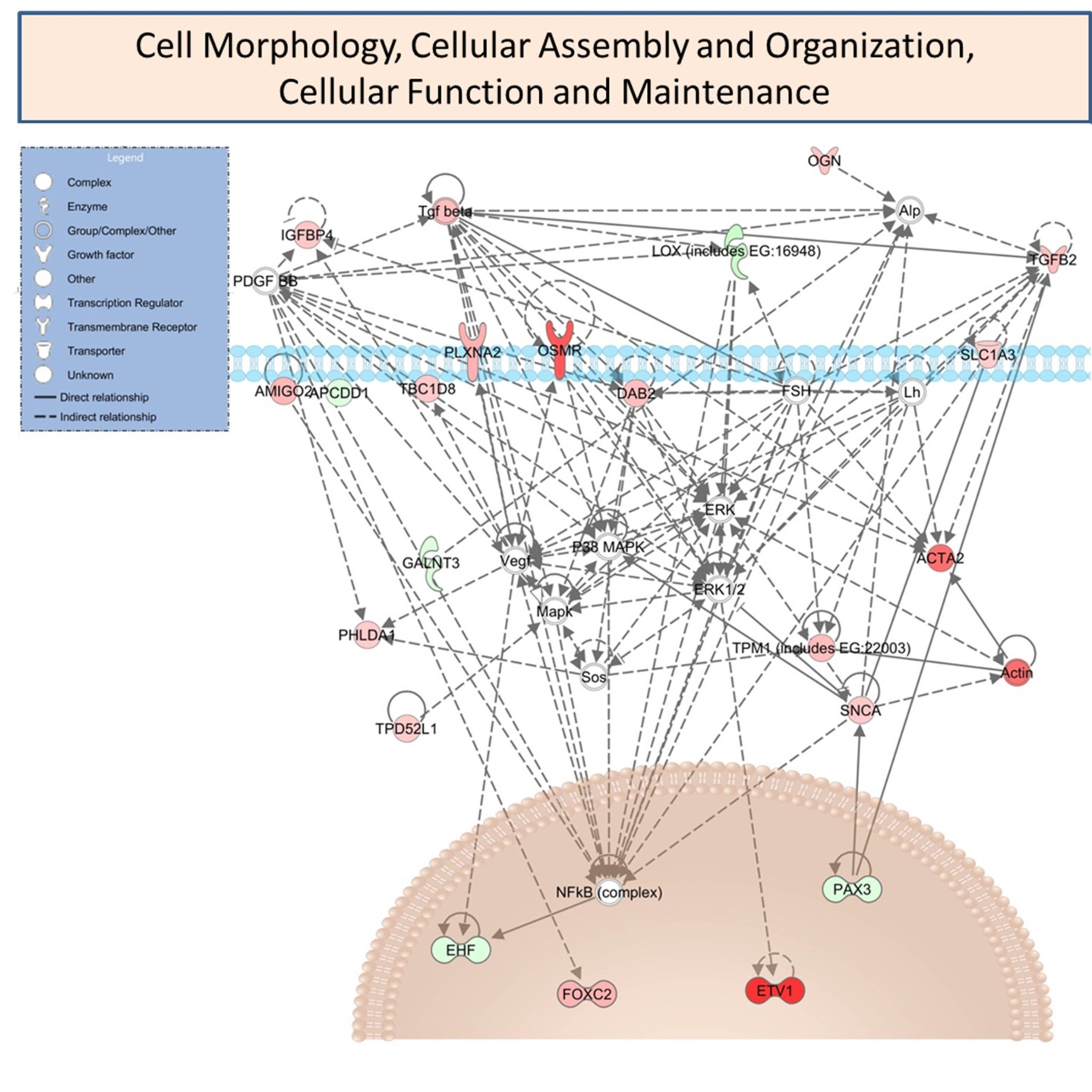
SUPPLEMEMTARY FIGURE 3: Top network identified by Ingenuity Pathway analysis after analysis of 48 commonly regulated genes in SAOS/LM5 and Dunn/LM8 cell systems. Red indicates down-regulation (fold change >2, fdr < 0.01) in both cell systems, green indicates up-regulation, with the color intensity indicating the degree of up- or down-regulation.

SUPPLEMENTARY TABLE 1: PCR primers used for validation of microarray data

Gene Primer sequence (5’-> 3’) Tm Product size (bp)

COL24A1 F GGCAGGCAGGGTTTAGCTGG 63.46 126

R ACGGATCCCAGGAATGCCCC 64.19

MAMDC2 F TGTGGCTTTGTGAACCGCTGG 63.97 120

R TGTAGTGGCCCAGTTCACTCTTGA 63.56

APCDD1 F AATGCCAAGAACCACGACCATGC 64.33 107

R AGGCCGATGGTCAGGTCTGC 64.40

EHF F AGCACAGTAGCAGAGTCACCT 60.83 88

R GAGTCCCTCTCGGGTTGTGC 62.51

PHOSPHO1 F CCACGAACGTGTGACGACGG 63.89 120

R TCCAACACCCTAGATAGGCAGCG 63.94

CCDC80 F GCTCACCCTCGGTAAAGAGAGA 61.21 134

R TGTGTAATCCAATGGTGGCTCAT 60.31

TGFB2 F CCTCCGAAACTGTCTGCCCA 62.11 85

R CCACAGAGCACCTGGGACTGT 64.06

ACTA2 F CCGGGACTAAGACGGGAATCCT 63.26 121

R CATCGTCCCCAGCAAAGCCG 64.08

SNCA F GCTCCCTCACGCCTTGCCTT 65.23 89

R TACACCACACTGTCGTCGAATGGC 65.13

PRUNE2 F CAGTGTGCTGGCGAGTGAAG 61.56 169

R ATGAGCCAGTTGCTCGGTGA 61.83

GAPDH F TGTTCGACAGTCAGCCGCAT 62.43 107

R GCCCAATACGACCAAATCCGT 61.02

F, forward primer; R, reverse primer; Tm, calculated melting temperature; bp, base pairs

SUPPLEMENTARY TABLE 2: Number of regulated (>2-fold; fdr < 0.01) probe sets enriched (fdr < 0.00001) at higher levels with significant nodes down to level 1

SUPPLEMENTARY TABLE 2: continued

SUPPLEMENTARY TABLE 3: Ranking of top bio functions following IPA analysis.


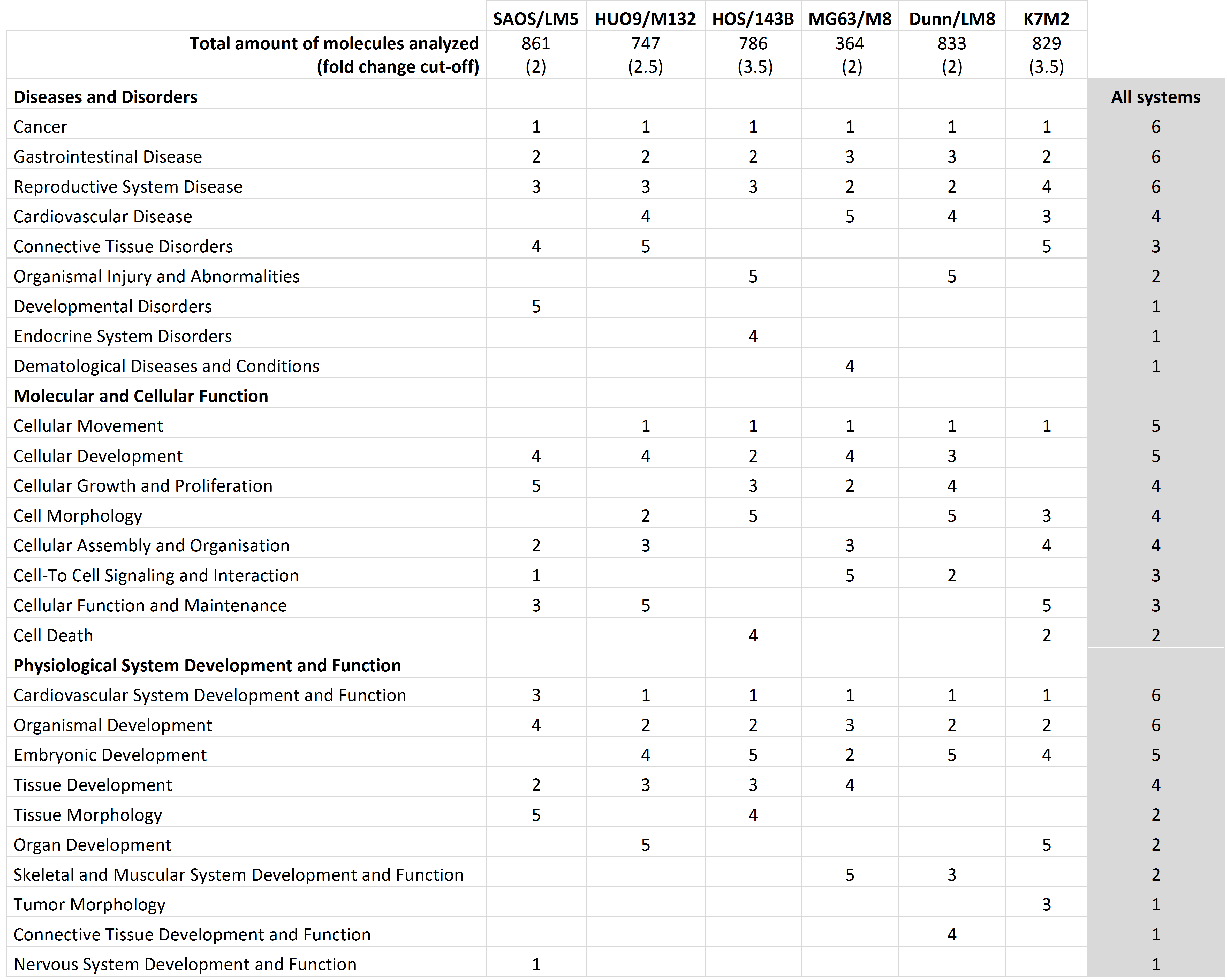


For each cell system, a cut-off was chosen such that comparable amounts of molecules were analyzed (in MG63/M8, a lower number of probe sets -521- were found to be significantly regulated, corresponding to a lower number -364- of recognized molecules). The top-5 bio functions, divided into the subcategories “diseases and disorders”, “molecular and cellular function” and “physiological system development and function” were compared among cell systems with the order displayed. Functions that were most commonly observed are shown first for each subcategory.

SUPPLEMENTARY TABLE 4: GO terms commonly up- or down-regulated in metastasis.

| **up-regulated** | **GO level** | **SAOS/LM5** | **Dunn/LM8** |
| --- | --- | --- | --- |
|  |  | **(probe sets (genes))** | |
| extracellular space | 3 | 47 (26) | 36 (24) |
| proteinaceous extracellular matrix | 3 | 31 (20) | 32 (24) |
| system development | 3 | 123 (83) | 119 (79) |
| binding | 1 | 415 (298) | 316 (218) |
| **down-regulated** |  |  |  |
| proteinaceous extracellular matrix | 3 | 34 (18) | 50 (26) |
| cell periphery | 3 | 160 (102) | 166 (109) |
| developmental process | 1 | 201 (126) | 199 (131) |
| multicellular organismal process | 1 | 228 (141) | 209 (140) |
| biological regulation | 1 | 279 (184) | 312 (211) |
| protein binding | 2 | 336 (204) | 366 (242) |
| carbohydrate binding | 2 | 25 (11) | 29 (17) |
